# The druggable schizophrenia genome: from repurposing opportunities to unexplored drug targets

**DOI:** 10.1038/s41525-022-00290-4

**Published:** 2022-03-25

**Authors:** Santiago G. Lago, Sabine Bahn

**Affiliations:** Department of Chemical Engineering and Biotechnology, University of Cambridge, Cambridge, UK

**Keywords:** Target identification, Schizophrenia, Pharmaceutics, Genome

## Abstract

There have been no new drugs for the treatment of schizophrenia in several decades and treatment resistance represents a major unmet clinical need. The drugs that exist are based on serendipitous clinical observations rather than an evidence-based understanding of disease pathophysiology. In the present review, we address these bottlenecks by integrating common, rare, and expression-related schizophrenia risk genes with knowledge of the druggability of the human genome as a whole. We highlight novel drug repurposing opportunities, clinical trial candidates which are supported by genetic evidence, and unexplored therapeutic opportunities in the lesser-known regions of the schizophrenia genome. By identifying translational gaps and opportunities across the schizophrenia disease space, we discuss a framework for translating increasingly well-powered genetic association studies into personalized treatments for schizophrenia and initiating the vital task of characterizing clinically relevant drug targets in underexplored regions of the human genome.

## Introduction

Schizophrenia is a complex and heterogeneous syndrome, affecting ~1% of the population and characterized by debilitating positive, negative, and cognitive symptoms in addition to severe comorbidities^1–3^. Despite the enormous burden on worldwide health including 1.9–2.8% of total years lived with disability^4,5^ and a 10–20 year reduction in life expectancy^3,6^, no drugs with novel mechanisms of action have emerged in the last three decades. Current antipsychotic medications only achieve full symptom remission in 15–25% of affected individuals^7,8^ and adverse side-effects such as weight gain, metabolic disturbances, over-sedation, extrapyramidal symptoms, and agranulocytosis^9,10^ are persistent problems. This is largely due to a lack of understanding of schizophrenia pathophysiology, incomplete characterization of the molecular targets of existing drugs, a scarcity of relevant preclinical models, and an inability to accurately predict treatment response as a result of disease heterogeneity^11–14^. In addition to these challenges, the increased rate of late-stage clinical trial failures and extended clinical development times for central nervous system (CNS) drug candidates^15,16^ has further dissuaded the pharmaceutical industry from pursuing novel drugs for schizophrenia.

In light of many of these difficulties, there has been renewed interest in recent years in drug repurposing, in other words, the identification of novel therapeutic indications for regulatory approved drugs^17^, in schizophrenia. The advantage of this strategy is that existing pharmacokinetic, dosing, toxicology and medicinal chemistry profiles of the drug candidates^18^ serve to expedite clinical trials in the new indication and reduce the costly attrition rate (~90%), associated with most novel drug entities^19^ particularly those aimed at neuropsychiatric indications. In many ways, this represents a return to the origins of schizophrenia drug discovery in the 1950s, when serendipitous clinical observation of the antipsychotic properties of drugs used in other indications, such as the pre-anesthetic chlorpromazine, laid the mechanistic foundation for the majority of monoaminergic drugs used today^10,13,20^. Although these monoaminergic compounds, which focus on differential dopamine and serotonin (5HT) receptor antagonism, have revolutionized the treatment of schizophrenia, many patient subgroups and symptom subdomains (e.g., negative symptoms and cognitive deficits) remain resistant to treatment^21,22^. A range of repurposing clinical trials^3,6,21,22^ have shown modest effect sizes in subgroups of patients. However low sample numbers, adjunctive administration protocols in chronically ill patients, and incomplete patient stratification have limited their applicability^23^.

The recent identification of genetic susceptibility loci for schizophrenia through large-scale genetic association studies suggests that each patient is likely to have a different combination of common but weak (weak effect on phenotype), or rare but penetrant (strong effect on phenotype), risk alleles^24–26^. These include over 138 schizophrenia risk loci identified in genome-wide association studies (GWAS) of common variation^26,27^, eight genome-wide significant loci affected by rare chromosomal copy number variants (CNV)^28^ and ultra-rare disruptive or de novo mutations which are enriched for gene sets involved in synaptic transmission^29,30^. Phenome-wide association studies (PheWAS)^31,32^, which correlate individual SNPs to multiple phenotypes, and transcriptome-wide association studies (TWAS)^33^, which integrate GWAS risk loci with genetic predictors of expression, have suggested further susceptibility genes. While these studies have provided support for existing schizophrenia drug targets, such as the dopamine 2 receptor (*DRD2*), they also offer an opportunity to identify drug targets which are not dependent on prior hypotheses of schizophrenia pathophysiology or the mechanisms of action of existing drugs. GWAS analyses in other disease indications have linked single-nucleotide polymorphisms (SNPs) in risk genes to widespread drug efficacy^34^. Examples include the interleukin-6 receptor gene, targeted by tocilizumab, in rheumatoid arthritis^35^ and the HMG-CoA reductase gene, targeted by statins, in conditions with elevated low-density lipoprotein cholesterol^34^.

One approach for prioritizing drug targets from disease association studies is to interpret the results in light of drug target annotation databases. The mechanism of action of many approved drugs is still subject to debate^36,37^ with comprehensive target lists more than doubling in recent times (266 in 2006^38^ vs. 667 in 2017^37^). This is due to improvements in drug–gene target mapping and the annotation of multi-target efficacies, complex subunits, and isoforms. Moreover, the fact that currently approved drugs only target a small fraction (3%) of the human proteome, relative to the estimated 15–35% of potentially druggable genes (i.e., genes which code for protein drug targets)^39,40^, has spurred efforts to characterize lesser studied human proteins and track their target development^41^. This has led to several market approvals in recent years including receptor deorphanization (i.e., identification of ligands for receptors which are predicted based on genetic or protein sequence information but for which endogenous ligands were previously unknown) for CNS targets *HCRTR1/2* and *S1PR1* genes to treat insomnia and multiple sclerosis respectively^41^. Nevertheless, recent analyses suggest that very little is known about ~40% of protein-coding genes in the human genome and that integration of diverse biomedical databases can provide a powerful tool for prioritizing underexplored drug targets^41^.

**Fig. 1:**
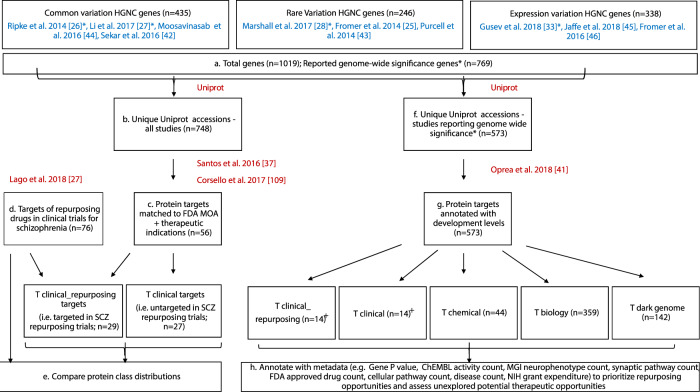
Workflow for annotation and cross-referencing of schizophrenia-associated genes and clinical repurposing drugs. Shows workflow described in Methods. Blue references are source studies of schizophrenia-associated genes. Red references represent cross-referencing resources used to annotate schizophrenia-associated genes and clinical trial drugs respectively. Uniprot refers to www.uniprot.org. Steps shown include the input of schizophrenia risk genes from source studies (**a**), identification of clinical repurposing targets suggested by genetic association studies (**b**, **c**), annotation of the targets of schizophrenia repurposing clinical trial drugs (**d**), comparison of the protein class distributions of targets suggested by genetic analyses and the schizophrenia clinical repurposing pipeline (**e**), annotation of genome-wide significant targets with IDG target development levels (**f**, **g**), prioritization of repurposing opportunities and unexplored therapeutic opportunities within the schizophrenia genome based on extended IDG target metadata (**h**). *Indicates studies reporting genome-wide significance, used for analyses focusing on genome-wide significance *P* values. ^+^ Indicates “T clinical” and “T clinical repurposing” target labels derived from steps (**b**–**d**).

Here we bring together the results of major studies exploring genetic association to schizophrenia to provide a consolidated list of risk and protective genes (Fig. 1). These include sources of common variants identified using GWAS and PheWAS, rare variants identified through CNV analysis and sequencing of disruptive or de novo exon mutations, and genes with altered tissue-specific expression identified using RNA-sequencing and TWAS analysis. We cross-reference the schizophrenia-associated genes with a comprehensive list of FDA-approved drug targets and indications to provide an update on immediate repurposing opportunities for schizophrenia. Moreover, we compare the protein class distribution of these potential repurposing targets with that of drugs currently in clinical repurposing trials for schizophrenia to identify which clinical trial candidates are supported by genetic evidence and which risk genes are yet to be targeted using approved drugs. Finally, we use extended compound annotation resources developed by the illuminating the Druggable Genome (IDG) initiative to prioritize the repurposing drug targets and elucidate unexplored therapeutic opportunities within the schizophrenia genome.

## Results and discussion

### Comparison of targets from genetic association studies and the clinical repurposing pipeline

**Fig. 2:**
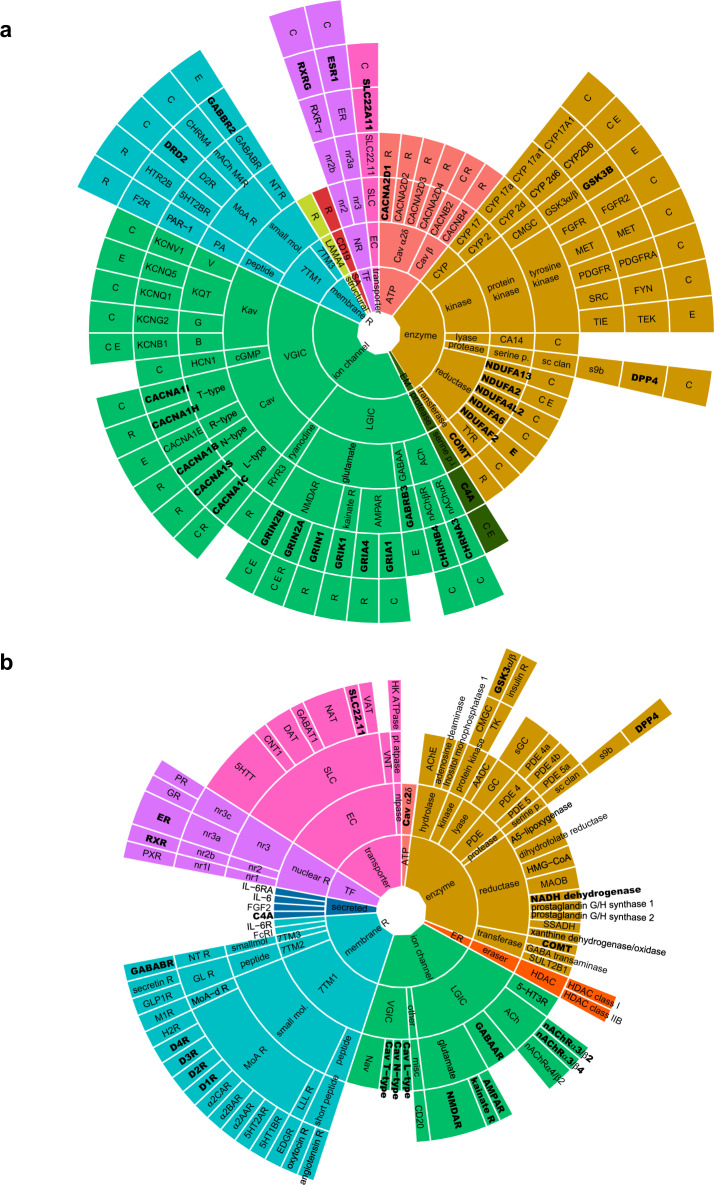
Protein class distribution of approved drug targets implicated by genetic association relative to repurposing clinical trials in schizophrenia. **a** Protein class distribution of 56 schizophrenia-associated genes (out of a total of 748)^25–28,33,42–46^ which are targeted by approved drugs (*n* = 187)^37^. Each segment concentrically shows the ChEMBL database protein target classification^37^, followed by the target gene (HUGO Gene Nomenclature Committee) and the source of genetic association in terms of analyses of common^26,27,42,44^ (C), rare^25,28,43^ (R), and gene expression^33,45,46^ (E) variation. Gene targets which match the targets of drugs in clinical repurposing trials for schizophrenia^23^ (*n* = 29) are shown in bold. **b** Protein class distribution of 76 human drug targets of approved drugs (*n* = 86) in clinical repurposing trials for schizophrenia. Each segment concentrically shows the ChEMBL protein target classification, followed by the ChEMBL drug target (individual proteins/protein complexes)^37^. The width of each drug target segment is proportional to the number of drugs associated with that target in clinical repurposing trials for schizophrenia. Some drugs have more than one target annotation (average 1.2). Drug targets which are supported by direct genetic evidence (*n* = 23) are shown in bold. Major protein target classes are colored as follows ion channel (green), membrane receptor (membrane R; turquoise), secreted (navy), structural (yellow), surface antigen (SA; red), transcription factor (TF; purple), transporter (pink), auxiliary transport protein (ATP; peach), enzyme (brown), enzyme modulator (EM; dark green), and epigenetic regulator (ER; orange). 7TM seven-pass-transmembrane domain receptors.

A total of, 748 unique genes were associated with schizophrenia by analysis of common, rare, and gene expression variation (Fig. 1; Methods; and Supplementary Data 1)^25–28,33,42–46^. From these, 56 genes mapped to the known protein targets of 187 approved drugs (termed “druggable” genes, Supplementary Data 2) as defined by the Santos et al. list of comprehensive FDA-approved drug targets^37^. Overall, 24, 17 and seven genes were unique to studies of common, rare, and expression variation respectively, with the remaining eight genes (*C4A*, *CACNA1C*, *CACNB2*, *CYP2D6*, *GRIN2A*, *GRIN2B*, *KCNB1*, and *NDUFA2*) represented in at least two types of analysis. Of the druggable genes, 27 (48%) have not yet been targeted in clinical trials for schizophrenia^23^ representing novel repurposing opportunities. Conversely, the 89 drugs listed in clinical repurposing trials for schizophrenia^23^ mapped to 76 unique human targets, of which 23 (26%) were supported by genetic evidence (Supplementary Data 3), suggesting that a significant proportion of the clinical repurposing pipeline is supported by direct genetic target associations, with a potentially larger share implicated by indirect or downstream targets^35^. Comparison of the protein class distributions of drug targets implicated by genetic studies (Fig. 2a) relative to repurposing clinical trials (Fig. 2b) revealed a series of established (widely tested in clinical trials), emerging (scarcely tested in clinical trials), and novel (untested in clinical trials) repurposing targets. In broad terms, ion channels were enriched among genetic targets (40% of targets) relative to clinical trials (22%), G-protein-coupled receptors (GPCRs), or seven-pass-transmembrane domain receptors (7TMs), transporters and nuclear transcription factors were reduced in the genetic results (9, 2, and 4% respectively) relative to drugs in clinical trials (17, 16, and 6% respectively). The enzyme class showed similar representation (29–30%) although with notable differences in composition.

### Established repurposing targets

Disease associations for subunits of nicotinic ACh receptor subtypes (e.g., *CHRNA3* and *CHRNB4*) and Glu receptor subtypes (e.g., NMDA receptor—*GRIN2A*, *GRIN2B*, and *GRIN1*; AMPA receptor—*GRIA1* and *GRIA4*; and kainite receptor—*GRIK1*) support the most extensive areas of repurposing activity, in terms of the number of clinical trials conducted, using glutamatergic (e.g., d-cycloserine and memantine) and cholinergic (e.g., galantamine, donepezil, and varenicline) cognitive enhancers^23^. Although clinical trials for these targets have met with mixed results^23^ the genetic data suggest that larger and well-controlled clinical trials employing key class derivatives might be warranted. In contrast, drugs related to monoaminergic, anti-inflammatory, metabolic, and hormonal mechanisms of action occupy a smaller target proportion in the genetic data relative to clinical trials. However, the clinical efficacy of subtypes of immune and metabolic compounds in subsets of patients, including the treatment of side-effects of medication, points to the fact that the genetically defined targets may not currently capture the full gamut of therapeutic possibilities.

### Emerging repurposing targets

Several target classes were strongly suggested by genetic evidence yet are relatively under-represented as direct targets in clinical trials. Principal among these were the CaV channel subunit genes which, when considering primary and auxiliary subunits, accounted for 21% of the genetic target space yet only 3% of clinical trial targets. L-type (*CACNA1C* and *CACNB2)* and T-type (*CACNA1I*) CaV channel subunits were among the most significant findings to emerge from GWAS studies in terms of reproducibility, significance, sole occupation of respective risk loci, and the fact that the primary risk SNPs for each gene are within the gene itself^26,27^. These findings are supported by further L-type (*CACNA1S* and *CACNB4*), T-type (*CACNA1H*), N-type (*CACNA1B*), R-type (*CACNA1E*), and auxiliary (*CACNA2D1–4*) CaV channel subunit associations from rare disruptive^43^ and de novo mutation^47^ exome sequencing and gene expression analysis^46^. Although the pathophysiological mechanisms involving CaV channels are not well understood, the fundamental role of CaV channels in neuronal signaling, gene transcription, and neurotransmitter trafficking makes them plausible targets^48,49^. This is supported by evidence that carriers of the principal L-type CaV channel risk allele show alterations in *CACNA1C* expression and fMRI connectivity in key schizophrenia-associated brain regions^50,51^.

Despite this evidence, only five approved CaV channel blockers are currently being tested for schizophrenia^23^, representing under 3% of current clinical trials. Interestingly, L-type CaV channel blockers were tested in several clinical repurposing trials for schizophrenia prior to the publication of the human genome. However, these clinical trials showed heterogeneous outcomes^35^. For example, verapamil improved positive symptoms in acute patients but showed no effects in chronic patients, nilvadipine improved negative symptoms with no change in positive symptoms in chronic patients and nifedipine had no effect in chronic patients but improved cognitive symptoms in patients with tardive dyskinesia^35^. The heterogeneous results obtained from these clinical trials could in part be explained by small sample sizes, difficulties in controlling for clinical variables, the inclusion of chronic treatment-resistant patients, and low brain penetrance of some of the compounds (e.g., verapamil)^35^. Phenotypic screening in schizophrenia patient samples has subsequently highlighted that 1,4-dihydropyridine (DHP) L-type CaV channel blockers with extended ester substitutions at the third position of the pyridine ring might be more therapeutically relevant derivatives^52^. Taken together these findings suggest that CaV channels, particularly L-type channels, with a wealth of approved drugs available, warrant further investigation as potential repurposing targets.

Another genetically associated target class which is under-represented in the clinical trial pipeline are the mitochondrial complex 1 (NADH dehydrogenase) subunits accounting for 9% of the genetic targets (*NDUFA13, NDUFA2, NDUFA4L2, NDUFA6*, and *NDUFAF2*) and only 1% of the clinical repurposing targets (NADH dehydrogenase). Although this is largely due to the implication of multiple subunits within a single protein complex, the clustering of risk variants from different genetic loci within the same complex nevertheless makes this a plausible drug target. This finding is supported by evidence of mitochondrial dysfunction in postmortem brain tissue^53^ and a positive correlation between blood mRNA expression of mitochondrial complex 1 subunits and psychotic symptoms^54^ in schizophrenia. However, metformin, which is the only drug annotation associated with mitochondrial complex 1, has proved ineffective in clinical trials for schizophrenia^55^ suggesting that targeting this protein may require other ligands^56^.

### Novel repurposing targets

Several drug targets which showed significant evidence of genetic association and were matched to approved drugs have not been tested in clinical trials, representing novel repurposing opportunities. Foremost among these were different subfamilies (B, G, KQT, V, and cGMP) of KV channels (*KCNB1, KCNG2, KCNQ1, KCNQ5, KCNV1*, and *HCN1*), further supported by mouse neurophenotypes and relative disease and pathway specificities (described below). *KCNB1* was notable as it was supported by both common variation and expression while *HCN1* had a higher level of ChEMBL activity and neurophenotype annotation. Limited evidence of KVs in schizophrenia includes increased *KCNB1* expression in neocortical developmental stages associated with schizophrenia^57^ and behavioral abnormalities reminiscent of schizophrenia in *KCNB1-AMIGO* functional knockout mice^58^. *HCN1* is strongly expressed in the dendrites of pyramidal cells in the cortex and hippocampus and is suggested to be involved in working memory and dendritic spine abnormalities in schizophrenia^59,60^. Importantly, KV channels work in concert with many of the aforementioned CaV channels to regulate neuronal excitability and neurotransmitter release^59^, representing a point of functional convergence between these genetic risk loci. They are also involved in physiological comorbidities of schizophrenia such as insulin resistance^61^. Approved drugs targeting KV risk genes include dalfampridine, ezogabine, guanidine, dronedarone, and ivabradine although the brain penetrance and side effect profiles of these compounds vary drastically. Additionally, drugs such as lamotrigine, gabapentin^35^, or the antiarrhythmic ibutilide, which was shown to ameliorate schizophrenia-associated cellular responses in phenotypic screens^52^, may serve to modulate KV function indirectly.

Other potentially novel repurposing opportunities supported by mouse neurophenotypes included cytochrome P450 enzymes, protein tyrosine kinases, mACh receptor, and B-cell surface marker. The cytochrome P450 enzymes are targeted by abiraterone and ketoconazole (*CYP17A1)* and quinidine (*CYP2D6*) respectively. *CYP17A1* is notable as a key enzyme required for the production of glucocorticoids and sex hormones, such as estrogen, which are linked to schizophrenia^62–64^, while *CYP2D6* is responsible for the dopamine synthesis in the brain^65^. Interestingly, *CYP2D6* is also involved in the metabolism of antipsychotic medications, with direct implications for their pharmacokinetics and clinical efficacy^66–68^, suggesting that compounds targeting *CYP2D6* may have potential as adjunctive medications. Protein tyrosine kinases (*FGFR2*, *MET*, *PDGFR*, *FYN*, and *TEK*) are notable as they indicate a highly druggable target class^37^ which has supported an increasing number drug approvals in recent years. However, toxicity and target characterization continue to represent significant hurdles. *FGF2* is a key mediator of neurogenesis and cortical patterning relevant to neurodevelopmental models of schizophrenia^69^ and both *FGFR2* and *FYN* interact with targets (PLC-γ1 and Src respectively) suggested by phenotypic screening^52^. Targeting the mACh receptor (*CHRM4*) is consistent with a renewed interest in selective mACh allosteric modulators for the treatment of schizophrenia^70^ and a significant increase in the grant funding behind *CHRM4* in recent years^23^. Finally, B-cell surface antigen *CD19* is notable with respect to functional abnormalities^71,72^ and genetic implications of B cells in schizophrenia^26^, although the targeting of this protein would require further elucidation given the importance of B cells in the immune response.

### Prioritization of repurposing opportunities in schizophrenia

**Fig. 3:**
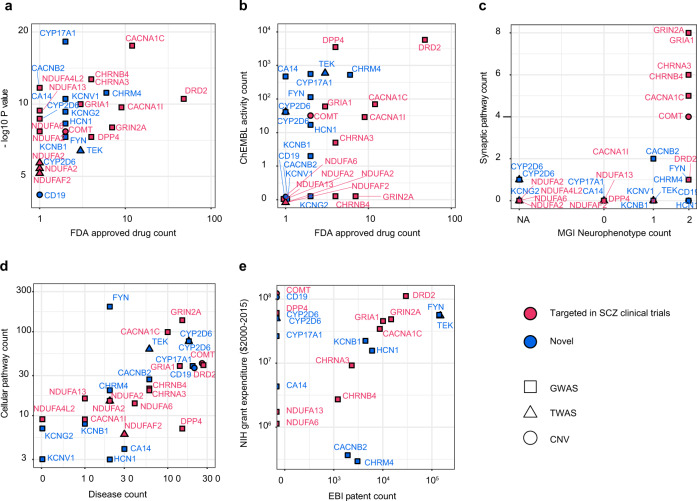
Prioritization of repurposing opportunities in schizophrenia. Shows genome-wide significant schizophrenia risk genes, from GWAS^26,27^ (*n* = 22, square), TWAS^33^ (*n* = 4, triangle), and CNV^28^ (*n* = 2, circle) reference studies, which are targeted by approved drugs^37^. Genes which have been targeted in clinical repurposing trials for schizophrenia^23^ are shown in red and novel repurposing opportunities are shown in blue. Axes comprise genome-wide significant *P* values from the respective studies cross-referenced with extended molecular drug target annotations for each gene^37,41^. Plots highlight gene ‘druggability’ in terms of **a** the number of FDA-approved drugs available and **b** abundance of chemical-target interaction data in the ChEMBL database, in addition to **c** CNS relevance in terms of the number of synaptic pathway annotations (Pathway Commons, KEGG, Reactome databases) and neurophenotypes resulting from orthologous gene mutations in mice (Mouse Genome Informatics database; NA—no phenotype data available, 0—non-neurophenotypes, 1—either MP:0003631 “nervous system phenotype” or MP:0005386 “behavior/neurological phenotype”, 2—both MP:0003631 “nervous system phenotype” or MP:0005386 “behavior/neurological phenotype”), **d** specificity in terms of total cellular pathway annotations (Pathway Commons, KEGG, Reactome databases) and disease associations and **e** commercial viability in terms of associated National Institutes of Health (NIH) grant expenditures (2000–2015) and total European Bioinformatics Institute (EMBL-EBI) patent counts. *P* values from different TWAS expression reference panels are represented as discrete points. Genes are labeled using standardized nomenclature (HUGO Gene Nomenclature Committee).

We sought to prioritize genetically supported repurposing opportunities in terms of practicality and novelty by cross-referencing a subset of druggable^37^ schizophrenia risk genes, from GWAS^26,27^ (*n* = 22), TWAS^33^ (*n* = 4), and CNV^28^ (*n* = 2) reference studies reporting genome-wide significance, with extended target annotation resources of the human genome curated by the IDG initiative (Fig. 1; Methods; and Supplementary Data 1)^41^. Comparison of the number of approved drugs available relative to the genome-wide association significance for each target per study (Fig. 3a) revealed that several of the most significant target genes with multiple drugs available (e.g., CaV channel subunits*- CACNA1C, CACNB2*, and C*ACNA1I*, nicotinic acetylcholine (nACh) receptor subunits—*CHRNA3* and *CHRNB4*, and GluR subunits GRIN2A and GRIA1) are already being targeted in clinical repurposing trials for schizophrenia. The gene with the highest number of approved drugs was *DRD2* reflecting existing approvals for antipsychotic drugs in schizophrenia.

Of the genes which are not currently direct clinical trial targets, *CYP17A1* was the most significant among the GWAS data and the muscarinic ACh receptor (*CHRM4*) had the largest number of available drugs. Other genes, which are not currently targeted in clinical trials, with intermediate significance and drug availability included KV channel subunits (*KCNV1, KCNG2, KCNB1*, and *HCN1*) and tyrosine kinases (*FYN* and *TEK*). To further prioritize the druggable genes, we assessed the amount of chemical-target interaction data in the ChEMBL database for each gene (Fig. 3b). This revealed several as yet untargeted genes which had greater functional target annotation than those which are already targeted in clinical trials for schizophrenia, including *CYP17A1, TEK, CHRM4*, *CA14*, and *FYN*.

Comparison of the relative implication of the druggable genes in synaptic pathways and orthologous nervous system or behavioral/neurological phenotypes (“neurophenotypes”) in transgenic mice (Fig. 3c), revealed that the majority of genes which are already targeted in schizophrenia have a high number of synaptic pathway annotations and mouse neurophenotypes. However, several of the untargeted risk genes such as *HCN1, FYN, CHRM4,* and *CD19* and to a lesser extent *CYP17A1, TEK, KCNB1*, and *KCNV1* were associated with mouse neurophenotypes, despite a low level of synaptic annotation, suggesting that these genes may be linked to pathophysiological mechanisms in the CNS which are either not fully characterized or fall beyond the scope of synaptic abnormalities. Exploration of genetic disease pleiotropy (i.e., the effect of single genes on multiple diseases) vs. pathway promiscuity (i.e., the involvement of single genes across multiple pathways) (Fig. 3d) suggested that while untargeted genes such as *CYP17A1, CYP2D6*, and *CD19* are relatively promiscuous in terms of their cellular pathway and physiological disease effects, other genes such as *KCNB1, KCNV1, KCNG2*, and *HCN1* are relatively specific to a subset of known pathways and schizophrenia. Finally, assessment of the drug development opportunities for currently untargeted druggable schizophrenia genes, by comparing grant expenditure with EBI patent counts (Fig. 3e), suggested that while patent numbers for the majority of the genes correlate with research expenditure, some genes such as *CYP17A1, CYP2D6*, and *CD19* have resulted in relatively few patents, despite large investment, whereas genes like *CHRM4* and *CACNB2* might be more patentable for the investment made.

### Unexplored therapeutic opportunities within the schizophrenia genome

**Fig. 4:**
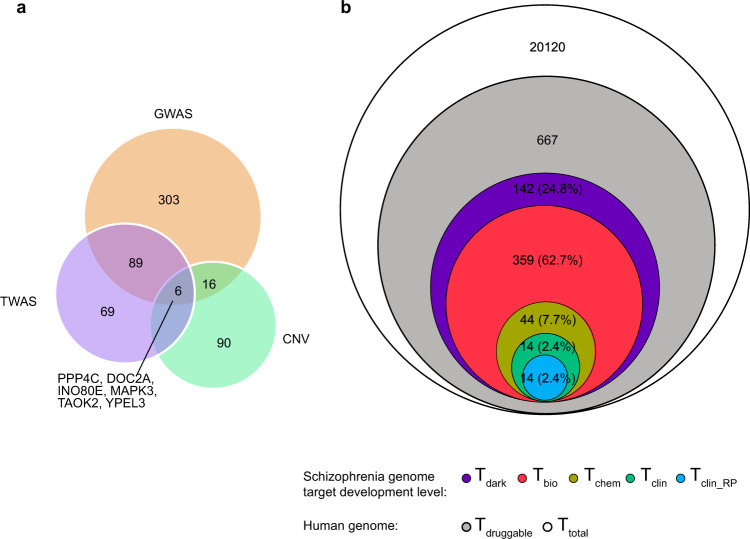
Distribution of genome-wide significant schizophrenia risk genes by data source and drug target development level. **a** Shows overlap between genome-wide significant schizophrenia risk genes (*n* = 573 unique), from GWAS^26,27^ (*n* = 414, orange), TWAS^33^ (*n* = 152, purple), and CNV^28^ (*n* = 112, green) reference studies. Genes shared between sources of schizophrenia-associated genetic variation data (*n* = 6) are labeled. Genes are labeled using standardized nomenclature (HUGO Gene Nomenclature Committee). **b** Schematic representation of the distribution of genome-wide significant schizophrenia risk genes across target development levels in terms of the number of genes and the % of total schizophrenia risk genes. Target development levels reflect the degree to which a gene target is characterized for therapeutic purposes in human disease indications and include T clinical (T_clin_; green)—targets linked to approved drug mechanisms of action, T chemical (T_chem_; yellow)—targets which bind small molecules with high potency, T biology (T_bio_; red)—targets with evidence of bioactivity and T dark genome (T_dark_; purple)—unexplored targets^41^. T clinical repurposing (T_clin_RP_; blue) reflects a subset of T_clin_ targets which additionally map to drugs which have been tested in clinical repurposing trials for schizophrenia^23^. Total druggable genes in the human genome, corresponding to human molecular targets of approved drugs across disease indications (T_druggable_; gray; *n* = 667)^37^, and the total number of genes in the human genome (T_total_; white; *n* = 20,120)^41^ are shown as background for comparison.

To prioritize genes associated with schizophrenia in terms of their potential druggability beyond immediate drug repurposing opportunities, we annotated all genome-wide significant schizophrenia risk genes (*n* = 573 unique), from GWAS^26,27^ (*n* = 414), TWAS^33^ (*n* = 152), and CNV^28^ (*n* = 112) reference studies (Fig. 4a), with target development categories from a recent comprehensive summary of unexplored therapeutic opportunities in the human genome curated by the IDG initiative (Fig. 1; Methods; and Supplementary Data 1)^41^. The IDG initiative categorizes protein-coding genes as four target types: T clinical (T_clin_)—targets linked to approved drug mechanisms of action (3%), T chemical (T_chem_)—targets which bind small molecules with high potency (6%), T biology (T_bio_)—targets with evidence of bioactivity (53%), and T dark genome (T_dark_)—unexplored targets (38%)^41^. Schizophrenia risk genes revealed an enrichment of potentially druggable targets associated with either preliminary chemical (T_chem_ 8%) or biological (T_bio_ 63%) evidence, in addition to the targets associated with approved drugs (T_clin_ and T_clin___RP_ 5%; described earlier) (Fig. 4b). This suggests that there is a host of potentially innovative drug targets for schizophrenia beyond those which currently match known drugs and is consistent with the concept that human disease mutations are more prevalent in core functional genes^73^.

Comparison of the sources of genetic evidence suggested that the majority of target space continues to be driven by common (GWAS) risk variants reflecting the larger statistical power and sample numbers in these studies. Overlap is greatest between GWAS and TWAS results (*n* = 89 genes) as GWAS risk loci are used to index TWAS SNP associations and the studies use overlapping sample sets. The data clearly highlights six genes (*PPP4C, DOC2A, INO80E, MAPK3, TAOK2*, and *YPEL3*) common to all datasets, as follow-up candidates. Interestingly, none of these genes mapped to approved drug targets, suggesting vital opportunities for clinical development. T_chem_ targets *MAPK3* and *TAOK2* are both serine/threonine-protein kinases which interact with the MAPK signaling cascade and are involved in the stability of the postsynaptic density (PSD), strongly implicated in schizophrenia^25,30,74,75^. *MAPK3* is further reported to be a regulatory trigger for the functional cassette involving *KCTD13* and *MVP* which regulates brain development and neuronal proliferation phenotypes^33^. T_bio_ genes *PPP4C* and *YPEL3* are involved in the regulation of histone acetylation^76^ and cellular senescence^77^, while T_dark_ genes *DOC2A* and *INO80E* are involved in spontaneous calcium-dependent neurotransmitter release^78^ and chromatin remodeling^79^, respectively. In addition to elucidating the role of these six genes in schizophrenia, the absence of highly specific ligands targeting these genes suggests that a greater toolbox of ligands to explore their therapeutic target validity in live cellular systems is required. In this respect, the ChEMBL activity annotations and knockout model organisms^33^ available for *MAPK3* and *TAOK2* are an amenable starting point for translational follow-up. Interestingly, while TWAS associations for most of these genes were linked to altered gene expression in the brain (*PPP4C, DOC2A, MAPK3*, and *TAOK2*), some risk alleles were associated with altered expression in the blood (*PPP4C* and *INO80E*) or adipose tissue (*PPP4C* and *YPLE3*). This highlights the fact that some schizophrenia risk genes show systemic expression alterations and raises questions about the genetic predisposition to side-effects of antipsychotic medication such as neutropenia and weight gain.

**Fig. 5:**
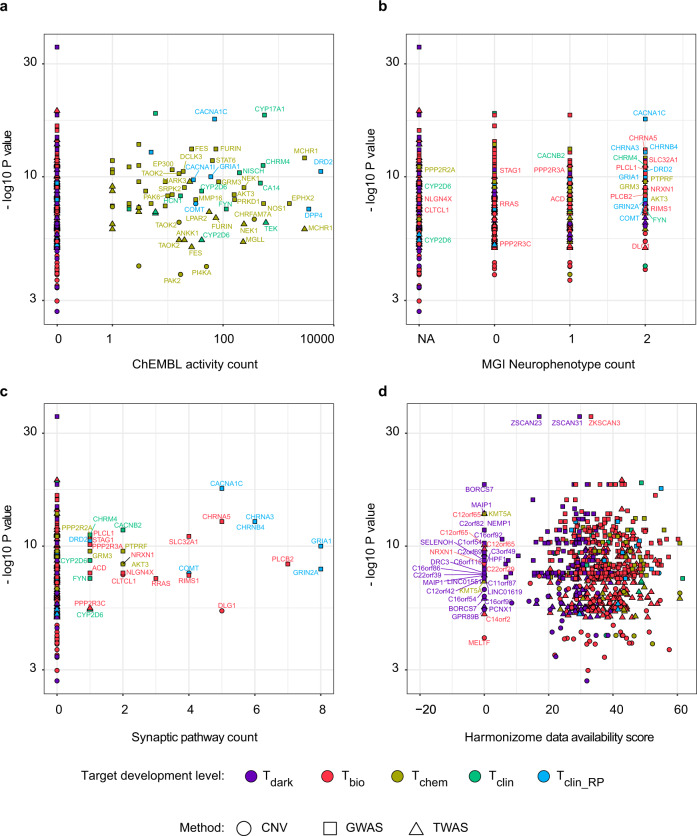
Unexplored therapeutic opportunities within the schizophrenia genome. Figure shows all genome-wide significant schizophrenia risk genes (*n* = 573 unique), from GWAS^26,27^ (*n* = 414, square), TWAS^33^ (*n* = 152, triangle), and CNV^28^ (*n* = 112, circle) reference studies. For each gene negatively transformed *P* values (−log *P* values) for genome-wide association to schizophrenia from the respective studies are cross-referenced with extended molecular drug target annotations^41^ to highlight therapeutic opportunities in terms of **a** “druggability” defined by abundance of chemical-target interaction data in the ChEMBL database, **b** CNS relevance defined by number of neurophenotypes resulting from orthologous gene mutations in mice (Mouse Genome Informatics database; NA—no phenotype data available, 0—non-neurophenotypes, 1—either MP:0003631 “nervous system phenotype” or MP:0005386 “behavior/neurological phenotype”, 2—both MP:0003631 “nervous system phenotype” and MP:0005386 “behavior/neurological phenotype”), **c** CNS relevance defined by number of synaptic pathway annotations (Pathway Commons, KEGG, Reactome databases), and **d** novelty in terms of the Harmonizome data availability score which integrates the cumulative probability of each gene (or protein product) occurring across 70 major publically available online resources and is an indicator of experimental information density^90^. *P* values from different TWAS expression reference panels are represented as discrete points. Target development levels reflect the degree to which a gene target is characterized for therapeutic purposes in human disease indications and include T clinical (T_clin_; green)—targets linked to approved drug mechanisms of action, T chemical (T_chem_; yellow)—targets which bind small molecules with high potency, T biology (T_bio_; red)—targets with evidence of bioactivity and T dark genome (T_dark_; purple)—unexplored targets^41^. T clinical repurposing (T_clin_RP_; blue) reflects a subset of T_clin_ targets which additionally map to drugs which have been tested in clinical repurposing trials for schizophrenia^23^. Genes are labeled using standardized nomenclature (HUGO Gene Nomenclature Committee). For representation only genes with ChEMBL activity >10 (**a**), at least one synaptic pathway annotation (**b**, **c**), or Harmonizome score <10 (**d**) are labeled.

Further dissection of genome-wide significant schizophrenia risk genes to reveal untapped potentially translatable drug targets showed a total of 60 genes with chemical-target interaction data in the ChEMBL database (Fig. 5a), 266 genes with associated mouse neurophenotypes (Fig. 5b), and 29 genes with synaptic pathway annotations (Fig. 5c and Supplementary Fig. 1). While, targets of clinical drugs (T_clin_), including those which are already in clinical repurposing trials for schizophrenia (T_clin_RP_), were well represented as a benchmark across these parameters, there were notable genes in the T_chem_ and T_bio_ categories which were significantly associated to the disease and had similar or even greater levels of annotation than the T_clin_/T_clin_RP_ targets, representing a second and third tier of potentially translatable targets respectively. These included T_chem_ genes (e.g., *MCHR1, MAPT, EPHX2, NOS1, CHRFAM7A, NEK1, MGLL, PRKD1*, and *AKT3*) with a wealth of ChEMBL chemical-target interaction data (including 14 genes with target-specific chemical ligands- Supplementary Data 1 ChEMBL selective compounds) and T_chem_ (e.g., *PTPRF, GRM3, GRM8*, and *AKT3*) and T_bio_ (e.g., *PLCB2, CHRNA5, DLG1, SLC32A1, RIMS1*, and *RRAS*) genes which had both mouse neurophenotype and synaptic pathway associations. Interestingly, while T_chem_ genes were abundant at the highest level of mouse neurophenotype association, T_bio_ genes (e.g., *PLCB2, CHRNA5*, and *DLG1*) dominated the synaptic pathway associations, indicating a paucity of ligands for synaptic proteins aside from neurotransmitter receptors.

Risk genes with high ChEMBL activity annotations represent more immediately translatable targets in the sense that they have a wealth of chemical ligand-binding and activity information which can be used to guide target development. For example, the risk gene *MCHR1*, which has not been targeted in schizophrenia, has a ChEMBL activity count comparable to that of the established schizophrenia target *DRD2*, including 4029 compounds and 5897 bioactivity registries. Moreover, MCHR1 signaling has been shown to modulate dopamine-related responses in mesocorticolimbic, but not nigrostriatal, dopaminergic pathways in animal models of schizophrenia^80^, suggesting that it may be a novel therapeutic target which avoids the extrapyramidal toxicity inherent to subclasses of antipsychotic drugs.

Risk genes with both synaptic pathway and neurophenotype annotations are more likely to be involved in pathophysiological mechanisms in the CNS and are practical in terms of the availability of animal models for behavioral testing and target validation. Among these, T_chem_ genes such as *GRM3* and *GRM8* are consistent with preliminary evidence of preclinical and clinical effects of metabotropic GluR modulation on symptoms of schizophrenia^81^, while genes such as *AKT3* are supported by altered expression of Akt isoforms in the brain and immune cells of schizophrenia patients^82^. Another T_chem_ candidate, receptor-type tyrosine-protein phosphatase F (*PTPRF*), is interesting in terms of its association with insulin resistance^83^, a feature which has recently been shown to correlate with the polygenic risk of schizophrenia and diminished treatment response in subgroups of first-episode patients^84^. Conversely, T_bio_ targets *PLCB2, PLCH2, PLCL1*, and *PLCL2* are notable in that related PLC isotypes have been linked to altered expression in the brain of schizophrenia patients postmortem^85^, schizophrenia-like behavioral abnormalities in knockout animal models^85,86^ and altered calcium flux responses in peripheral blood cells of drug-naïve schizophrenia patients^52^. Moreover, *DLG1* and *NRXN1* are hub proteins in synaptic and ARC-complex networks strongly associated with schizophrenia^87^, while *RIMS1* and *SLC32A1* are involved in synaptic vesicle exocytosis^88^ and vesicular GABA reuptake, respectively^89^. Taken together these findings suggest that the cross-examination of T_chem_ and T_bio_ targets with metadata such as ChEMBL activity scores or synapse and neurophenotype annotations can highlight translatable gene targets which might be mechanistically plausible and practical despite lower levels of clinical development in the context of schizophrenia.

To assess how the targets were distributed in terms of novelty we mapped the risk genes against an independent composite score (Harmonizome data availability (HDA) score) which integrates the cumulative probability of each protein occurring across 70 publically available online resources and is an indicator of the experimental information density associated with the protein (Fig. 5d)^90^. These resources include experimental data such as biomolecular interactions, expression in cell lines and tissues, genetic associations with the knockout mouse or human phenotypes, and changes in expression after drug treatment derived from large “-omics” repositories and publications. As expected, HDA scores were greatest for T_clin_ and T_clin_RP_ genes followed by T_chem_, T_bio_, and T_dark_ genes respectively, reflecting a gradient of novelty which is inversely related to the HDA score. Interestingly, schizophrenia risk genes were distributed in a bimodal manner across this target space. Well-documented risk genes such as *CACNA1* and *GRIN2A* led the first wave with high HDA scores followed by a range of T_chem_ and T_bio_ targets.

However, towards the other end of the scale, the results revealed a subgroup of genes (*n* = 27; Supplementary Fig. 1), comprised primarily of T_dark_ annotations, which had low HDA scores and about which relatively little is known. Notably, several of these genes (e.g., *ZSCAN23, ZSCAN31, ZKSCAN3*, and *BORCS7*) were more significantly associated with schizophrenia than those under clinical development suggesting that they might represent drug targets which require further functional characterization. Recent data suggest that lysosomal trafficking protein *BORCS7*, in conjunction with T_bio_ target *AS3MT*, might alter early neuronal differentiation in subgroups of schizophrenia patients^91^. Conversely, DNA-binding zinc-finger proteins *ZSCAN23* and *ZSCAN31* are representative of a larger group of significantly associated transcription factors (*n* = 37) which are scarcely characterized in the context of schizophrenia. Although transcription factors have previously been less druggable than other protein families^41^, recent zebrafish phenotypic screens have underlined the importance of specific T_dark_ transcription factors (e.g., ZNF536) in functional phenotypes relevant to schizophrenia, such as the development of forebrain neurons implicated in social behavior and stress^92^. Moreover, the present data suggest up to 15 underexplored schizophrenia-associated transcription factors with orthologous mouse neurophenotypes (Supplementary Data 1). This suggests that systematic efforts to characterize and target lesser-known transcription factors or their druggable downstream targets are warranted.

Closer examination of schizophrenia-linked T_dark_ proteins as a whole revealed nine genes with associated mouse neurophenotypes of which three (*DOC2A*, *INO80E*, and *HIRIP3*) were supported by more than one analysis method (GWAS, TWAS, and CNV; Supplementary Fig. 2a) and relatively specific for neurophenotypes compared to other mouse phenotypes (Supplementary Fig. 2b). Underexplored genes such as *DOC2A* and *INO80E*, involved in spontaneous calcium-dependent neurotransmitter release and chromatin remodeling respectively, are clear candidates for follow-up. Likewise, genes, such as *C22orf39*, specific for neurophenotypes and about which scarcely anything is known to indicate a potentially relevant knowledge gap.

### Translational challenges and perspective

The gene-target associations discussed represent an initial shortlist of known susceptibility genes across the existing target annotation space, with an emphasis on identifying potential low-hanging fruits. However, the translation of genetic variants into drug discovery opportunities poses several challenges with relevance to future work.

First, the translational of gene-target associations into clinical compounds depends on whether the gene has a disease-modifying effect, whether the compound implicated evokes a change in the activity of the target in the desired direction and, vitally, the pharmacokinetics and safety of any resulting drug candidate in humans. While these steps fall beyond the scope of the current review, which focuses on target hypothesis generation, they form a crucial framework for follow-up studies.

Second, the majority of genetic risk loci for schizophrenia are yet to be defined. Polygenic risk profile scores based on GWAS risk loci, currently account for 3–8% of disease liability for schizophrenia and individual risk loci often have relatively small effect sizes^26,27^. While theoretical projections suggest that RPS might be able to explain up to 25–33% of disease liability, this remains considerably less than the 65–80% heritability observed in family and monozygotic twin studies^3,93^. Moreover, it is becoming apparent that large increases in sample size are required to achieve modest increases in the number of common risk loci identified^94^, with many of these likely to be of low odds ratio or low frequency within the population. Although susceptibility loci with modest odds ratios, for example, *DRD2* (odds ratio 1.08) which is targeted by current antipsychotics, can represent valid drug targets it is likely that many of the risk alleles already identified will continue to represent the most robust genetic risk factors.

Moreover, it is difficult to isolate the causative genomic variants and genes within putative susceptibility loci. Several of the implicated loci are too broad to distinguish genes which are genuine drug target candidates from genes which are simply in linkage disequilibrium with the true causative variants. For example, the extended major histocompatibility complex (MHC) which is the most significant PGC-SCZ GWAS locus, encompassing hundreds of genes, is excluded from most GWAS drug target analyses due to inconclusive identification of causative variants, with the exception of genes coding for C4A^95^. The exclusion of a large number of immune-related genes in this region may contribute to the lack of genetic evidence for immune-targeted repurposing candidates. Conversely, it is currently unknown whether many of the genes included in this study are causative and corrections for gene size, effect size, and multiple testing are restricted to the studies of origin. In this respect, the current study represents a parallel framework for evaluating whether a gene is druggable and consequently a relevant lead to follow-up in terms of fine mapping. High-resolution mapping, using deep-coverage and long-read whole-genome sequencing, may improve identification of causative genomic variants, especially rare variants within non-coding regions, and help to answer more complex questions such as structural variation, variants in repetitive DNA, and phasing^96^.

Third, the most poignant challenge is understanding the functional implications of putative risk genes and how they interact to elicit the altered cellular and organismal phenotypes associated with the disease. The majority of risk SNPs reside in intergenic regions, introns, or correspond to synonymous mutations. Therefore it is hard to ascertain how a given risk polymorphism affects gene expression or protein function and whether an agonist or antagonist is required to target the disease phenotype. While TWAS and brain RNA-seq studies have shed light on this, expression profiles can vary depending on the reference tissue type used^50,51^. Conversely, expression quantitative trait loci (eQTL) catalogs require greater curation to accurately ascertain tissue specificity for a given genomic variant^97^. Projects such as PsychENCODE^98^ and Genotype-Tissue Expression (GTEx)^99^ aim to address this by exploring the pathophysiological relationship between non-coding regulatory elements and gene expression patterns in different brain regions from patients and controls. Moreover, the most relevant drug targets for schizophrenia might not be directly implicated risk loci (each of which likely has a relatively small or nonspecific impact on the phenotype), but instead hub proteins with disease-modifying effects on multiple risk genes within a cellular network. Although protein–protein interaction maps of schizophrenia susceptibility loci have begun to address this there remain challenges in terms of reconstruction and prioritization of hub proteins, as seen from the high number of FDA-approved candidate compounds implicated by some studies^100^.

Fourth, in terms of drug repurposing, the matching of risk genes to known drug targets is only as complete as the drug target annotation on which it is based. The Santos et al. molecular drug target list^37^ used to annotate schizophrenia risk genes, is based largely on the mechanism of action annotations from FDA-approved drug labels and primary literature. It is therefore robust in identifying low-hanging fruit for repurposing found at the intersection between well-characterized clinical targets and genetic risk loci. However, in many ways, the list is conservative in terms of defining the full repurposing opportunities related to genetic variation in schizophrenia. For example, analyses using the Drug–Gene Interaction Database (DGIdb)^40^ and the Psychoactive Drug Screening Database (Ki DB)^101^, which look more broadly at drug–gene interactions beyond FDA mechanisms of action, suggest further interactions with potassium channels (*KCNN3* and *KCNJ13*), cytochrome P450 enzymes (*CYP26B1* and *CYP21A2*), and ACh receptor subunits (*CHRNA5*), in addition to other novel targets. However, the therapeutic and pharmacokinetic implications of many of these interactions are less well documented and in some cases may represent adverse reactions. In either case, the matching of risk genes to approved drug targets represents a heuristic means of shortlisting repurposing drug candidates, which subsequently require closer examination in terms of the direction of pharmacological effect, dosing, contraindications, and therapeutic intent. In this respect, there is also an urgent need to develop a standardized ontology of drug activity for computational mining^102^ and to account for direct or indirect targets which are not associated with FDA-approved mechanisms of action. Although FDA-approved drug targets annotations have increased drastically in recent years^37,38^, this still reflects a small portion (3%) of the human proteome and falls considerably short of the 15–35% of genes which are theoretically druggable^39,40^. It is therefore vital to track the development level of protein targets beyond approved drug matches to identify both imminent potential repurposing opportunities, such as T_chem_ proteins which are targeted in clinical trials for novel drugs.

Finally, although the study of lesser-known genes, such as the T_dark_ proteins associated with schizophrenia, continues to face challenges, such as confirmation bias and risk aversion, several initiatives are underway to address this knowledge deficit. These include the Monarch Initiative^103^, which integrates clinical data with model organisms to identify phenotypically relevant cross-species disease models, or the International Mouse Phenotyping Consortium (IMPC)^73^, which aims to phenotype knockout mouse lines for up to 20,000 human orthologue genes. Recent data from the IMPC shows that approximately one-third of single-gene knockout models so far have at least one significant neurophenotype observation, many of which overlap with Online Mendelian Inheritance in Man (OMIM)^104^, GWAS Catalog^105^, and DISEASES^106^ databases. Functional phenotypic screening in schizophrenia patient-derived cellular models^107^, such as primary peripheral blood cells ex vivo^52,72^ or iPSC-derived neurons^108^, or risk-gene knockout model organisms^92^ have also been used to functionally characterize lesser-known genes. Importantly these approaches support the screening of novel compound or drug repurposing libraries, such as the Repurposing Hub^109^ or National Centre for Advancing Translational Sciences (NCATS)^110^ libraries, so that gene sets which are strongly associated with the disease yet have a paucity of ligand-binding information, such as PSD proteins or subsets of transcription factors, might be targeted. These efforts are further supported by improvements in the diversity of molecular probes as represented by the NIH Molecular Libraries Initiative^111^ or signature matching between drug and disease transcriptome profiles^112^.

The boundaries of the druggable genome are constantly evolving in terms of the number of target proteins and the depth of target annotation, suggesting that many more therapeutic targets are possible across the human disease space. Integration of genetic data with systematic evidence-based protein target annotation is a powerful tool for prioritizing hypotheses of drug targets in schizophrenia, ranging from new potential repurposing opportunities such as those represented by members of the voltage-gated potassium channel or cytochrome P450 families to relatively unexplored genes with orthologous neurological and behavioral phenotypes, such as *INO80E* and *DOC2A*, found at the intersection between common, rare and expression genetic risk variants. This approach provides a valuable means to evaluate the results of previous clinical trials in addition to providing a framework for addressing genetic heterogeneity in future drug discovery efforts. Together with new experimental techniques which address the daunting, yet essential, the task of illuminating lesser studied proteins in the human genome, this approach serves to drive the identification of mechanistically diverse potential drug candidates and support much-needed personalized therapeutic improvements in treatment-resistant patient populations or symptom domains of schizophrenia.

## Methods

### Comparison of targets from genetic association studies and the clinical repurposing pipeline

Schizophrenia risk genes which matched HUGO Gene Nomenclature Committee (HGNC) references (*n* = 1019) were compiled from the largest and most recent reference analyses exploring sources of common (*n* = 435)^26,27,42,44^, rare (*n* = 246)^25,28,43^, and gene expression (*n* = 338)^33,45,46^, genetic variation associated with schizophrenia (Supplementary Data 1 and Fig. 1a). Gene IDs and corresponding metadata (e.g., reference SNPs, analysis methods) from the source studies were matched to unique UniProt accession numbers (*n* = 748; Fig. 1b) to provide a list of schizophrenia-associated protein targets. UniProt accessions (protein targets) were annotated with drug target efficacy information for approved drugs (e.g., drug name, molecule type, FDA mechanism of action (MOA), ChEMBL target ID, ChEMBL protein target classification, FDA approval date, and anatomical therapeutic chemical classification (ATC) codes)^37^, and therapeutic disease indications^109^ to determine protein targets which are targeted by approved drugs and the respective therapeutic indications of these drugs (Fig. 1c). In parallel, the same drug target efficacy information^37^ was used to map the protein targets of drugs in the clinical repurposing pipeline for schizophrenia (described in detail below; Fig. 1d). The ChEMBL protein target classifications of repurposing targets suggested by genetic analyses and targets in the schizophrenia clinical repurposing pipeline were compared (Fig. 1e) to identify areas of overlap (i.e., targets which are, “T clinical_repurposing” (*n* = 29), or are not, “T clinical” (*n* = 27), under development in the schizophrenia repurposing pipeline. The later “T clinical” represents novel repurposing opportunities).

### Annotation of schizophrenia repurposing clinical trial drugs

Drugs explored in clinical repurposing trials for schizophrenia (*n* = 89 including ketamine, ClinicalTrials.gov)^23^ were annotated with drug target efficacy information for approved drugs (e.g., molecule type, mechanism of action, UniProt accession, ChEMBL target ID, ChEMBL protein target classification, FDA approval date, and anatomical therapeutic chemical classification code)^37^ and therapeutic disease indications^109^ (Fig. 1d). Drugs (*n* = 7) listed as having primary microbial targets were cross-referenced with the mechanism of action annotations in Drug Bank and corresponding human Uniprot accessions and ChEMBL IDs were added to the drug profile where relevant (minocycline, pyrimethamine, amantadine, and ceftriaxone). Anti-microbial drugs without human annotations in Santos et al.^37^ or Drug Bank (artemisinin, cysteine, and cycloserine) were excluded. Uniprot accessions were used to assess overlap between targets for drugs in schizophrenia clinical repurposing trials and schizophrenia risk genes. For drugs with multiple target annotations (*n* = 20), up to two protein target classification entries were selected for each drug, based on matching to schizophrenia risk genes (if applicable), primary therapeutic relevance (Drug Bank mechanism of action), and mechanistic diversity across the drug set. Drugs with multiple subunit annotations within the same target protein complex were consolidated to reflect a single protein target classification.

### Prioritization of repurposing opportunities and unexplored therapeutic opportunities within the schizophrenia genome

A subset of studies, reporting genome-wide significant *P* values^26–28,33^(Fig. 1a*), were used for prioritization of repurposing opportunities in schizophrenia and exploring therapeutic opportunities within the schizophrenia genome. These comprised 769 schizophrenia-associated genes, derived from common (GWAS; *n* = 416), rare (CNV; *n* = 112), and gene expression (TWAS; *n* = 241) genetic variation studies (Supplementary Data 1), which matched to 573 unique UniProt accession numbers (Fig. 1f). UniProt accessions were annotated with protein target development levels (TDLs) from genomic, proteomic, chemical, and disease-related human genome data repositories curated by the Illuminating the Druggable Genome (IDG) Knowledge Management Center^41^ (Fig. 1g). These included: “T clinical”—targets linked to approved drug mechanisms of action, “T chemical”—targets which bind small molecules with high potency, “T biology”—targets with evidence of bioactivity, and “T dark genome”—unexplored targets. An additional category was created for proteins targeted in clinical repurposing trials for schizophrenia, “T clinical_repurposing”. TDLs for targets previously matched to FDA mechanisms of action in the repurposing analysis (described above) were labeled accordingly as “T clinical_repurposing” and “T clinical” (Fig. 1g^**+**^). Uniprot accessions for each target were also annotated with extended metadata curated by the IDG Knowledge Management Center (e.g., HUGO Gene Nomenclature gene name, protein family, publication metrics, antibody count, HarmonizomeDAS score, Gene Ontology count, OMIM phenotypes, grant funding, pathway count, disease count, murine orthologue phenotype count, ChEMBL activity count, and PANTHER classification)^41^. Neurophenotypes resulting from orthologous gene mutations in mice were defined using IDG data based on the Mouse Genome Informatics database using search terms MP:0003631 “nervous system phenotype” and MP:0005386 “behavior/neurological phenotype”. The distribution of genome-wide significant schizophrenia targets was then examined across the TDL space and cross-referenced with the IDG extended metadata to prioritize both potential repurposing targets and unexplored therapeutic opportunities (Fig. 1h). For cross-referencing, genome-wide significance *P* values for schizophrenia-associated genes are reported as in the study of origin. Therefore *P* values are only comparable within targets from the same analysis type (i.e., common, rare, and expression variation).

### Reporting Summary

Further information on research design is available in the Nature Research Reporting Summary linked to this article.

## Supplementary information

Supplementary information

Supplementary Data 1

Supplementary Data 2

Supplementary Data 3

Reporting Summary Checklist

## Data Availability

All data generated or analysed during this study, including aggregate data, are included in this published article (and its supplementary information files). Source data is available as UniProt accession codes (www.uniprot.org) with references in Supplementary Data 1 and descriptions in the Methods.

## Code availability

Code is available from the corresponding author on reasonable request.
