# Supplementary material for: The druggable schizophrenia genome: from repurposing opportunities to unexplored drug targets
Source: NPJ Genom Med. 2022 Mar 25;7:25. doi: 10.1038/s41525-022-00290-4 (PMC8956592; doi:10.1038/s41525-022-00290-4)
Supplement: Supplementary file 1 — Supplementary information [file 41525_2022_290_MOESM1_ESM.pdf]

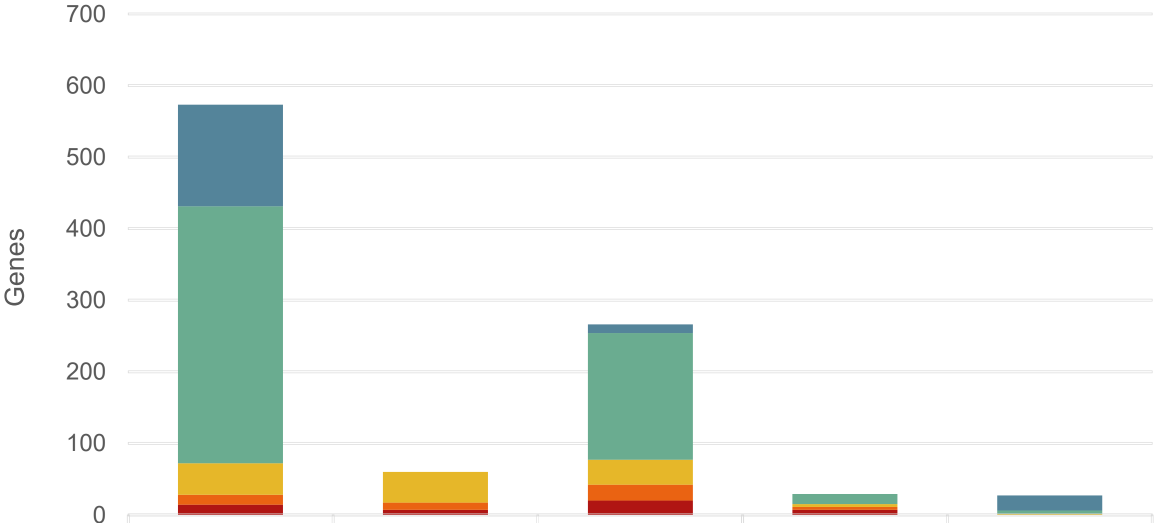

	Total	ChEMBL activity	Neurophenotype	Synapse pathway	Harmonizome<1
Tdark	142	0	12	0	21
Tbio	359	0	177	14	5
Tchem	44	43	35	4	1
Tclin	14	10	22	4	0
Tclin.RP	14	7	20	7	0

Supplementary Figure 1 Distribution of genome-wide significant schizophrenia risk genes across target development levels and metadata. Figure shows genome-wide significant schizophrenia risk genes (n=573 unique), from GWAS(26, 27) (n=414), TWAS(33) (n=152) and CNV(28) (n=112) reference studies, cross-referenced with extended molecular drug target annotations(41) to highlight therapeutic opportunities in terms of (a) 'druggability' defined by at least one chemical-target interaction annotation in the ChEMBL database, (b) CNS relevance defined by at least one neurophenotype resulting from orthologous gene mutations in mice (Mouse Genome Informatics database; MP:0003631 'nervous system phenotype' or MP:0005386 'behavior/neurological phenotype'), (c) CNS relevance defined by at least one synaptic pathway annotation (Pathway Commons, KEGG, Reactome databases) and (d) novelty in terms low (<1) Harmonizome data availability scores(90). Target development levels reflect the degree to which a gene target is characterized for therapeutic purposes in human disease indications and include T clinical (Tclin; orange) - targets linked to approved drug mechanisms of action, T chemical (Tchem; yellow) - targets which bind small molecules with high potency, T biology (Tbio; aqua) - targets with evidence of bioactivity and T dark genome (Tdark; blue) - unexplored targets(41). T clinical repurposing (Tclin_RP; red) reflects a subset of Tclin targets which additionally map to drugs which have been tested in clinical repurposing trials for schizophrenia(23).

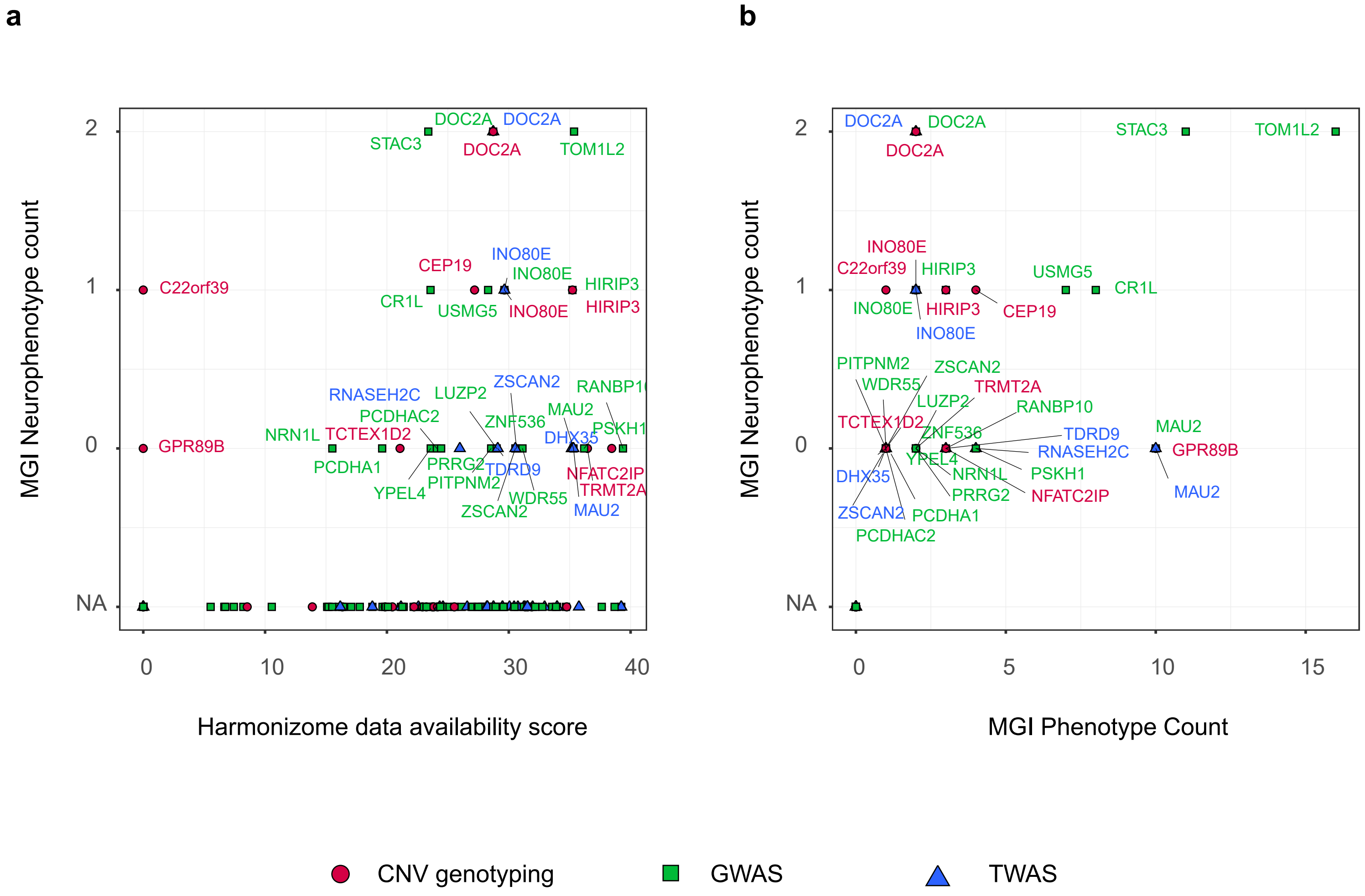

Supplementary Figure 2 Prioritization of schizophrenia-associated Tdark targets across orthologous mouse phenotypes and Harmonizome data availability scores. Figure shows genome-wide significant schizophrenia risk genes (n=142), from GWAS(26, 27) (square), TWAS(33) (triangle) and CNV(28) (circle) reference studies, which are part of the part of the Tdark target development category(41) and represent unexplored targets in the human genome. Plots show CNS relevance for each gene defined by number of neurophenotypes resulting from orthologous gene mutations in mice (Mouse Genome Informatics database; NA - no phenotype data available, 0 - non-neurophenotypes, 1 - either MP:0003631 ‘nervous system phenotype’ or MP:0005386 ‘behavior/neurological phenotype’, 2 - both MP:0003631 ‘nervous system phenotype’ or MP:0005386 ‘behavior/neurological phenotype’) against (a) novelty in terms of the Harmonizome data availability score, which integrates the cumulative probability of each gene (or protein product) occurring across 70 major publically available online resources and is an indicator of experimental information density(90), and (b) number of total orthologous mouse phenotypes. Genes are labelled using standardized nomenclature (HUGO Gene Nomenclature Committee).

SUPPLEMENTARY DATA LEGENDS

Supplementary Data 1 Target annotation of schizophrenia risk genes. Shows schizophrenia risk gene entries (n=1019; n=748 unique) from reference studies of common, rare and gene expression genetic variation(*25–28*, *33*, *42–46*). Genes are labelled using standardized nomenclature (HUGO Gene Nomenclature Committee; column A) and annotated with source information (risk variant type, study and method; columns B-U), UniProt accession numbers and synonyms (columns V-AF), drug target efficacy data for approved drugs (columns AG-AR)(*37*), therapeutic disease indications(columns AS-AV)(*109*), target overlap with drugs in schizophrenia clinical repurposing trials (columns AW-AX)(*23*) and extended genomic, proteomic, chemical and disease-related protein target annotations curated by the Illuminating the Druggable Genome (IDG) Knowledge Management Center (columns AY-DI)(*41*). Multiple entries for a given protein target (e.g. multiple drugs targeting the same protein) are concatenated within the same cell. Columns T, U, AI, CI, CN-CP and CS are derived from adjacent columns. Columns M-S refer solely to TWAS data(*33*). Abbreviations include schizophrenia (SCZ), HUGO Gene Nomenclature Committee (HGNC), single nucleotide polymorphism (SNP), genome-wide association study (GWAS), transcriptome-wide association study (TWAS), negative log10 P value (nlog10.Pvalue), genome-wide significant (GWS), Anatomical Therapeutic Chemical Classification (ATC), Target Central Resource Database (TCRD), target development level (TDL), TDL including schizophrenia repurposing targets in clinical trials (TDL.RP), National Center for Biotechnology Information (NCBI), Drug Target Ontology (DTO), Gene Reference Into Function (GeneRIF), antibody (Ab), Gene Ontology (GO), National Institutes of Health (NIH), European Bioinformatics Institute (EBI), Harmonizome data availability score (HarmonizomeDAS), Online Mendelian Inheritance in Man database (OMIM), Jackson Laboratory Mouse Genome Informatics (JAX.MGI), Protein Analysis Through Evolutionary Relationships (PANTHER), Broad Institute Human L1000 epsilon (L1000), RCSB Protein Data Bank ID (PDB), Gene Expression Omnibus accession GSE2685 (GSE2685). TDL.RP categories include T clinical (Tclin) - targets linked to approved drug mechanisms of action, T clinical repurposing (Tclin_RP) - targets linked to approved drug mechanisms of action which have been targeted in clinical repurposing trials for schizophrenia, T chemical (Tchem) - targets which bind small molecules with high potency, T biology (Tbio) - targets with evidence of bioactivity and T dark genome (Tdark) - unexplored targets.

Supplementary Data 2 Repurposing opportunities in schizophrenia. Shows schizophrenia risk genes (n=56 unique) from all reference studies(*25–28*, *33*, *42–46*) which matched the targets of approved drugs(*37*). Genes are labelled using standardized nomenclature (HUGO Gene Nomenclature Committee) and annotated with source information (risk variant type, study and method) in addition

to drug target metadata (protein name, protein class description, drug count, drug names, mechanism of action, target ChEMBL ID, molecule type, Anatomical Therapeutic Chemical Classification code)(*37*), therapeutic disease indications(*109*) and target overlap with drugs in schizophrenia clinical repurposing trials (targeted SCZ clinical trial (Y/N) and SCZ clinical trial drug)(*23*). Data shown represents a subset of data from Supplementary Data 1 and 3.

Supplementary Data 3 Target annotation of approved drugs in clinical repurposing trials for schizophrenia. Shows drugs targeting human proteins in clinical repurposing trials for schizophrenia (n=86; ClinicalTrials.gov; columns A-C)(*23*) annotated with human drug target efficacy information for approved drugs (columns D-N)(*37*), protein target overlap with schizophrenia risk genes (column O) and therapeutic disease indications (columns P and Q)(*109*). Drugs having primary microbial targets for which additional human target annotations were cross-referenced in Drug Bank are denoted in column N.
